# Examining Access to Digital Technology by Race and Ethnicity and Child Health Status Among Chicago Families

**DOI:** 10.1001/jamanetworkopen.2022.28992

**Published:** 2022-08-26

**Authors:** Kristin Kan, Nia Heard-Garris, Anne Bendelow, Lu Morales, Marquita W. Lewis-Thames, Matthew M. Davis, Marie Heffernan

**Affiliations:** 1 Division of Advanced General Pediatrics and Primary Care, Department of Pediatrics, Ann & Robert H. Lurie Children’s Hospital of Chicago, Northwestern University, Chicago, Illinois; 2 Data Analytics and Reporting, Ann & Robert H. Lurie Children’s Hospital of Chicago, Chicago, Illinois; 3 Smith Child Health Outcomes, Research, and Evaluation Center, Stanley Manne Children’s Research Institute, Ann & Robert H. Lurie Children’s Hospital of Chicago, Chicago, Illinois; 4 Department of Medical Social Science, Center for Community Health, Feinberg School of Medicine, Northwestern University, Chicago, Illinois

## Abstract

This cross-sectional study characterizes the type and quality of digital access among racially and ethnically as well as socioeconomically diverse households with children in Chicago.

## Introduction

During the COVID-19 pandemic, use of digital technology has become more widespread for routine activities for children (eg, medical visits, remote learning), perpetuating concerns about the US socioeconomic “digital divide.”^1^ In 2021, 19.7% of parents reported using telemedicine for their child.^2^ However, for health care to adequately address children’s health needs, defining the quality, access, and use of digital technology among families is essential. We characterize the type and quality of digital access among racially and ethnically as well as socioeconomically diverse households in Chicago and the associations with child and household characteristics.

## Methods

Data collection for this cross-sectional study occurred from May 24 to July 29, 2021, through a panel survey of parents in English and Spanish via web and telephone. Eligibility criteria included being 18 years or older, the parent of at least 1 child living in the household, and a Chicago resident. Respondents were recruited from probability samples (1134 of 2280 eligible responses [49.7%]) (eMethods in the Supplement). The institutional review board at Lurie Children’s Hospital approved the study, and participants provided electronic consent. This study followed the STROBE reporting guideline.

Survey questions addressed type of device, perceived internet speed, internet costs, and concerns about affordability (eTable in the Supplement). Demographic characteristics and child health status were collected. Self-reported parent race and ethnicity were categorized as Asian and other race or ethnicity (which were combined owing to small sample size), Hispanic or Latinx, non-Hispanic Black (hereinafter Black), and non-Hispanic White (hereinafter White). All percentages and analyses were population weighted; a 2-sided *P* < .05 was considered significant. Differences in digital access by child health status and demographic characteristics were examined using χ^2^ tests. Analyses were performed in SAS software, version 9.4 (SAS Institute, Inc). Multivariable logistic regression examined the likelihood of having high-speed internet with race and ethnicity and household income as covariates.

## Results

Data from 1620 Chicago parents (58.5% [95% CI, 55.0%-62.1%] women and 41.5% [95% CI, 37.9%-45.0%] men; 71.5% [95% CI, 68.2%-74.8%] aged 26-45 years) were collected. In terms of race and ethnicity, 9.5% (95% CI, 7.5%-11.6%) were Asian or other, 38.1% (95% CI, 34.7%-41.4%) were Hispanic or Latinx, 21.4% (95% CI, 18.5%-24.3%) were Black, and 31.0% (95% CI, 28.0%-34.0%) were White. Most parents (90.5% [95% CI, 88.6%-92.4%]) reported having a desktop or laptop at home, and 76.7% (95% CI, 73.7%-79.7%) reported having reliable high-speed internet (Table 1). Black and Hispanic or Latinx parents were less likely to have reliable, high-speed internet (76.0% [95% CI, 69.5%-82.6%] and 64.5% [95% CI, 58.8%-70.2%], respectively) vs White parents (88.8% [95% CI, 85.3%-92.3%]; *P* < .001) (Table 2). Child health status was not associated with having high-speed internet. In the multivariable model, Hispanic or Latinx parents were less likely to have high-speed internet vs White parents (odds ratio, 0.45 [95% CI, 0.28-0.73]) after controlling for household income. There was no significant difference for having high-speed internet between Black and White parents (odds ratio, 0.92 [95% CI, 0.53-1.61]).

**Table 1.** Characteristics of Survey Respondents

Characteristic	Unweighted No. of participants (N = 1620)	Weighted % (95% CI)
Sex^a^		
Female	1159	58.5 (55.0-62.1)
Male	455	41.5 (37.9-45.0)
Parent’s age, y		
18-25	118	4.0 (2.9-5.1)
26-45	1211	71.5 (68.2-74.8)
≥46	291	24.5 (21.3-27.7)
Federal poverty level^a^		
<100%	230	17.7 (15.1-20.4)
100%-399%	725	46.8 (43.4-50.2)
≥400%	663	35.4 (32.3-38.6)
Parent race and ethnicity		
Asian and other race or ethnicity^b^	147	9.5 (7.5-11.6)
Hispanic or Latinx	590	38.1 (34.7-41.4)
Non-Hispanic Black	290	21.4 (18.5-24.3)
Non-Hispanic White	593	31.0 (28.0-34.0)
Parent educational level^a^		
High school or less	309	36.6 (33.1-40.2)
Some college or technical school	369	23.4 (20.7-26.2)
College graduate or more	925	39.9 (36.7-43.1)
Age of children in household		
All 0-5 y	355	18.7 (16.2-21.2)
≥1 Child 11 y or older	853	56.9 (53.6-60.3)
All others	412	24.3 (21.4-27.2)
Household child health status		
All children have excellent or very good health	NA	85.4 (83.0-87.8)
≥1 Child with good, fair, or poor health	NA	14.6 (12.2-17.0)
Types of devices^c^		
Desktop or laptop	1460	90.5 (88.6-92.4)
Smartphone	1533	93.5 (91.5-95.4)
Tablet or portable wireless computer	1204	72.8 (69.7-75.9)
Other	22	1.7 (0.6-2.7)
None	6	0.3 (0.0-0.8)
Has reliable high-speed internet		
Yes	1299	76.7 (73.7-79.7)
No	321	23.3 (20.2-26.3)
Cost of internet per month, $^a^		
<40.00	258	18.4 (16.5-20.3)
40.00-59.99	367	23.2 (21.2-25.3)
60.00-79.99	363	21.1 (19.2-23.2)
80.00-99.99	281	16.1 (14.4-18.0)
≥100.00	220	13.9 (12.2-15.6)
Not sure	117	7.4 (6.2-8.7)
Worried about paying for internet^a^		
Yes	343	23.9 (20.9-26.9)
No	1265	76.1 (73.1-79.1)

Abbreviation: NA, not applicable.

^a^ There were nonresponses for the following demographic variables: sex (n = 6), federal poverty level (n = 2), parent education level (n = 17), cost of internet (n = 14), and worried about paying for internet (n = 12).

^b^ Owing to small sample sizes, respondents from Asian and other race or ethnicity were combined, including individuals who self-identified as Asian Indian, Native American, more than 1 race, or other.

^c^ Can select more than 1 response.

**Table 2.** Association of Digital Access With Child Health Status and Race and Ethnicity

Variable	Digital access, weighted % (95% CI)					
	Has reliable high-speed internet			Worried about paying for internet		
	Yes	No	*P* value	Yes	No	*P* value
Child health status						
All children had excellent or very good health	78.0 (74.8-81.2)	22.0 (18.8-25.2)	.09	23.2 (20.0-26.5)	76.8 (73.5-80.0)	.27
≥1 Child with good, fair, or poor health	70.0 (61.4-78.6)	30.0 (21.4-38.6)		28.1 (20.1-36.1)	71.9 (63.9-79.9)	
Race and ethnicity						
Asian or other race or ethnicity	87.8 (80.9-94.8)	12.2 (5.2-19.1)	<.001	16.3 (7.5-25.2)	83.7 (74.8-92.5)	<.001
Hispanic or Latinx	64.5 (58.8-70.2)	35.5 (29.8-41.2)		32.0 (26.5-37.5)	68.0 (62.5-73.5)	
Non-Hispanic Black	76.0 (69.5-82.6)	24.0 (17.4-30.5)		28.6 (21.4-35.7)	71.4 (64.3-78.6)	
Non-Hispanic White	88.8 (85.3-92.3)	11.2 (7.7-14.7)		13.1 (9.6-16.7)	86.9 (83.3-90.4)	
Federal poverty level						
<100%	53.2 (44.8-61.7)	46.8 (38.3-55.2)	<.001	NA	NA	NA
100%-399%	74.0 (69.3-78.6)	26.0 (21.4-30.7)		NA	NA	
≥400%	91.8 (88.8-94.9)	8.2 (5.1-11.2)		NA	NA	

Abbreviation: NA, not applicable.

## Discussion

The findings of this population-weighted cross-sectional survey of Chicago families suggest that disparities in digital access were associated with race and ethnicity as well as income but were not associated with child health status. After adjusting for income, the disparity in access to high-speed internet persisted for Hispanic or Latinx but not Black participants. This may be attributed to racial and ethnic residential segregation, because Chicago ranks high nationally on segregation indices, suggesting that broadband services may be limited in neighborhoods with residents who are members of racial or ethnic minority groups.^3^ Previous studies have found that Hispanic or Latinx adults are more likely than Black or White adults to report having smartphone internet only.^4^ Barriers to access might include affordability and real or perceived low demand, discouraging internet carriers to invest in broadband infrastructure where Hispanic/Latinx families live.^5^

Study limitations include the generalizability to a national population, participants’ representativeness by income of Chicago families, and possible underrepresentation of participants with limited internet access. These findings are important for the design and implementation of digital health services and policies in childrens’ health care, ensuring that specific populations are not overlooked.^6^

## Notes

Supplement. **eMethods.** Parent Panel Survey, Recruitment, and Analysis **eTable.** Digital Access Survey Questions and Categorical Responses
eReferences
