# Supplementary material for: Examining Access to Digital Technology by Race and Ethnicity and Child Health Status Among Chicago Families
Source: JAMA Netw Open. 2022 Aug 26;5(8):e2228992. doi: 10.1001/jamanetworkopen.2022.28992 (PMC9419010; doi:10.1001/jamanetworkopen.2022.28992)
Supplement: Supplement. — eMethods. Parent Panel Survey, Recruitment, and Analysis eTable. Digital Access Survey Questions and Categorical Responses eReferences [file jamanetwopen-e2228992-s001.pdf]

Supplementary Online Content

Kan K, Heard-Garris N, Bendelow A, et al. Examining access to digital technology by race and ethnicity and child health status among Chicago families. *JAMA Netw Open*. 2022;5(8):e2228992. doi:10.1001/jamanetworkopen.2022.28992

eMethods. Parent Panel Survey, Recruitment, and Analysis
eTable. Digital Access Survey Questions and Categorical Responses
eReferences

This supplementary material has been provided by the authors to give readers additional information about their work.

© 2022 Kan K et al. *JAMA Network Open*.

eMethods. Parent Panel Survey, Recruitment, and Analysis

Voices of Child Health in Chicago – Parent Panel Survey

The Voices of Child Health in Chicago (VOCHIC) Parent Panel Survey is a triannual survey of Chicago parents about child, adolescent, and family health and well-being. Parents in the panel are from all 77 neighborhoods in Chicago. Parents indicated their preferred method for completing surveys (online or phone) during their initial survey with the panel, and the majority (97%) of parents completed the survey online. Web-based surveys have increased in popularity over the last decade as phone survey response rates have declined.[1] Respondents were compensated $5 to $15, based on whether they were first-time participants.

Recruitment and Sampling Methodology

Parent respondents are recruited to participate in the Voices of Child Health in Chicago (VOCHIC) Parent Panel through one of four mechanisms implemented by NORC at the University of Chicago: 1) probability-based panels (i.e., VOCHIC Parent Panel and NORC's AmeriSpeak panel) 2) address-based sampling, 3) nonprobability respondent-driven sampling, and 4) established online nonprobability survey panels (Dynata and Lucid panels). Responses from nonprobability samples were included to ensure sufficient sample size, which has been shown to be a cost-effective method to supplement probability-based samples.[2]

Data Weighting and Analysis

For the probability-based samples, base sampling weights are adjusted to account for nonresponse via a raking ratio method to American Community Survey (ACS) 18+ Chicago parents population totals associated with the following topline socio-demographic characteristics: age, sex, education, race/ethnicity, and Census Division, and the following socio-demographic interactions: age × gender, age × race/ethnicity, and race/ethnicity × gender.

For the nonprobability samples, we explicitly account for potential bias using NORC's True North calibration[3], a hybrid calibration approach developed at NORC based on small area estimation methods. The purpose of TrueNorth calibration is to adjust the weights for the nonprobability sample to bring weighted distributions of the nonprobability sample in line with the population distribution for characteristics correlated with the survey variables. Such calibration adjustments help to reduce potential bias, yielding more accurate population estimates. Pairwise deletion was used for missing data.

© 2022 Kan K et al. *JAMA Network Open*.

eTable. Digital Access Survey Questions and Categorical Responses

Question Type	Survey Questions	Response Options
Types of devices	At your home, do you or any members of your household own or use any of the following types of devices? *(select all that apply)*	Desktop/laptop<br>Smartphone<br>Table or other portable wireless computer<br>Other
Perceived internet speed	How would you describe the internet that you most use at home?	Reliable, high-speed internet [Yes]<br>Reliable but slow internet [No]<br>Not reliable internet [No]<br>Do not have internet at home [No]
Costs for internet	How much is your internet per month?	Less than $40 per month<br>$40-$59.99<br>$60-$79.99<br>$80-$99.99<br>More than $100<br>I'm not sure
Concern paying for internet	Did any of the following happen over the past few months? … You worried about being able to pay for your internet connection	Yes<br>No

© 2022 Kan K et al. *JAMA Network Open*.

eReferences

1. Lavakras PJ, Benson G, Blumberg S, et al. Report from the AAPOR Task Force on: The future of U.S. general population telephone survey research. American Association of Public Opinion Research. 2017. https://www.aapor.org/Education-Resources/Reports/The-Future-Of-U-S-General-Population-Telephone-Sur.aspx Accessed 7/4/2022.

2. Elliott MN, Haviland AM. Use of a web-based convenience sample to supplement a probability sample. Surv Methodol. 2007;33(2):211-215.

3. NORC at the University of Chicago. NORC's TrueNorth Calibration tool for probability and nonprobability samples: New Version 2.0 even more effective. August 2021.

© 2022 Kan K et al. *JAMA Network Open*.
